# Mediterranean diet and asthma in adults: a multicentre case-control study in a general population sample

**DOI:** 10.1186/s12931-025-03484-3

**Published:** 2026-01-13

**Authors:** Maria Carelli, Jessica Miotti, Maria Elisabetta Zanolin, Maria Beatrice Bilò, Roberto Bono, Mattia Cominacini, Angelo Guido Corsico, Pietro Pirina, Ernesto Crisafulli, Marcello Ferrari, Lucia Cazzoletti

**Affiliations:** 1 Cardiology Unit, IRCCS Azienda Ospedaliero-Universitaria di Bologna, Bologna, Italy; 2 Department of Diagnostics and Public Health, Unit of Epidemiology and Medical Statistics, University of Verona, Verona, Italy; 3 DISCLIMO - Università Politecnica delle Marche, Ancona, Italy; 4 Allergy Unit - Azienda Ospedaliero-Universitaria delle Marche, Ancona, Italy; 5 Department of Public Health and Pediatrics, University of Turin, Turin, Italy; 6 Department of Engineering for Innovation Medicine, University of Verona, Verona, Italy; 7 Division of Respiratory Diseases, IRCCS Policlinico San Matteo Foundation, Pavia, Italy; 8 Department of Medicine, Surgery and Pharmacy, Respiratory Unit, University of Sassari, Sassari, Italy; 9 Department of Medicine, Respiratory Medicine Unit, University of Verona and Azienda Ospedaliera Universitaria Integrata of Verona, Verona, Italy

**Keywords:** Asthma, Mediterranean diet, Neutrophils, Oxidative stress, Rhinitis

## Abstract

**Background:**

The relationship between diet, respiratory diseases and the mechanisms underlying their association are still poorly understood. In an adult sample, we investigated the associations between diet quality evaluated by two dietary scores (Italian Mediterranean Index (IMI), Modified Mediterranean Score (MDS)) and asthma and rhinitis prevalence. Additionally, total and differential white blood cells count, as inflammation indicators and two markers of oxidative stress, 8-iso-prostaglandin F_2α_ (8-isoprostane) and 8-oxo-dihydro-deoxyguanosine (8-OHdG), were measured.

**Methods:**

A total of 1183 subjects (aged 20–84) in the frame of a population-based multi-case-control study were studied: 186 with current asthma (CA), 103 with past asthma (PA), 445 with rhinitis (RN) and 449 controls. Food intake was assessed by using the EPIC Food Frequency Questionnaire. 8-OHdG and 8-isoprostane were measured on spot urine and standardized by creatinine.

**Results:**

A higher adherence to IMI was associated with a lower risk of CA (RRR: 0.50; 95%CI: 0.31;0.81; *p* = 0.005), but not PA or RN. In CA a higher adherence to the Mediterranean diet (MD) was associated with a lower neutrophil percentage out of total leukocytes (p for trend = 0.022 and *p* = 0.014 for IMI and MDS, respectively). Urinary 8-isoprostane was not associated with the MD adherence in any of the groups. In subjects with CA, those who were highly adherent according to MDS had higher 8-OH-dG urinary concentrations than non-adherents (*p* = 0.040) and in subjects with PA, they had lower 8-isoprostane than non-adherents (*p* = 0.026).

**Conclusions:**

Our findings suggest that the MD might be a protecting factor for asthma, but not for rhinitis. The link between diet and asthma is probably the reduction in chronic inflammation, whereas oxidative stress does not seem to influence this relationship.

**Supplementary Information:**

The online version contains supplementary material available at 10.1186/s12931-025-03484-3.

## Background

Although asthma is one of the most common chronic lung diseases [1, 2], it remains poorly understood and preventive strategies are lacking. Over recent decades, the prevalence of asthma, rhinitis and allergies has increased, due to a potential consequence of environmental and lifestyle changes [3]. One of the probable culprits is diet quality modification [2], characterized by a higher consumption of processed and refined foods, and a lower intake of fresh vegetables and fruits [4].

A trend towards a lower prevalence of asthma in southern Europe and in the Mediterranean regions that share a similar dietary pattern, was recognized by the International Study of Asthma and Allergies in Childhood and by the European Community Respiratory Health Survey [5, 6]. Furthermore, epidemiological studies indicate that the Mediterranean diet (MD) might be protective against asthma in children [7, 8], although other studies could not confirm these results [9].

Studies investigating the relationship between MD and asthma in adults are limited, and those investigating the association between MD and asthma symptoms or control provided inadequate levels of evidence [10, 11]. A variety of limitations must be considered in these studies. First of all, many of them focus on specific foods or nutrients rather than on holistic dietary patterns [4], not considering that foods are a complex combination of many nutrients and bioactive substances that interact with each other [12]. Another obstacle is the inconsistency of the MD definitions utilized, since there are several different diets around the Mediterranean basin [13, 14]. Another point to clarify derives [15] from the question of whether it is the diet per se or some other social or economic factors linked to the Mediterranean lifestyle which favourably drive the association.

Regarding the potential mechanisms, it has been reported that a diet with a high content of fruits, vegetables and legumes is associated with a lower level of inflammation [16], a pathophysiological mechanism that possibly connects behavioral factors to the risk of developing chronic diseases [17]. Furthermore, it has been hypothesized that the protective effect against asthma/atopy of MD is linked to its high antioxidants content [18].

In this multicase-control study on adults from the Italian general population, we investigated the association between asthma, rhinitis and the MD adherence, as evaluated by the Italian Mediterranean Index (IMI) [19, 20] and the Modified Mediterranean Score (MDS) [21, 22]. Another intent was to investigate the mechanisms at the basis of the diet/asthma and diet/rhinitis association, focusing on the urinary levels of 8-isoprostane and 8-oxo-dihydro-deoxyguanosine, as markers of oxidative stress, and on circulating white blood cells (WBC) as inflammation indicators.

## Materials and methods

### Study design

Subjects aged 21 to 86 years, enrolled in the Genetic and Environmental interaction in Respiratory Diseases (GEIRD) project, participated in the study. This population-based multi-case-control, multi**-**center study [23] had 2 stages. In stage 1, a screening questionnaire on respiratory symptoms was administered to random samples from the general population or pre-existing cohorts (the Italian Study on Asthma in Young Adults [24] and the Italian arm of ECRHS) [6, 25]). In the second stage, all the subjects with symptoms suggestive of asthma, chronic bronchitis, COPD, and samples of subjects with rhinitis and subjects without symptoms were invited to undergo a clinical interview, lung function tests, and methacholine test for phenotyping. Urine and blood samples were collected during the clinical visit. For each participant, the time of collection, the number of smoked cigarettes, and any medications taken immediately before collection (in the case of current asthma) were recorded.

All measurement protocols adhered to international guidelines [23]. Ethical approval was obtained in each centre and informed consent was obtained from each participant. This analysis includes subjects recruited in the centers of Pavia, Torino, Sassari, Ancona and Verona, who completed the food frequency questionnaire (FFQ) European Investigation into Cancer and Nutrition (EPIC) [26].

### Lung function test

Forced expiratory volume in 1 s (FEV1) and forced vital capacity (FVC) were measured according to the ATS criteria [27]. FEV1% predicted and the lower limit of normal (LLN) for the FEV1/FVC were calculated according to Quanjer [28]. Spirometry was repeated 10 min after administering 400 µg salbutamol in subjects with FEV1/FVC < 70% or < LLN. Subjects with FEV1/FVC ≥ 70% and ≥ LLN underwent the methacholine challenge test [6]. A positive test resulted from a 20% FEV1 decrease at a cumulative dose of methacholine ≤ 1 mg (PD20 ≤ 1 mg).

### Biomarkers measurement

The container with the urine sample was kept at 4 °C for up to 24 h, then aliquoted into 1 mL cryovials and frozen at − 80 °C until analysis. Spot urine samples were processed at the National Institute of Cancer Research laboratory (Genova, Italy). 8-OHdG (8-oxo-dihydro-deoxyguanosine) and 8-isoprostane (8-iso-prostaglandin F2α) were quantified by using competitive enzyme-linked immunosorbent assay (ELISA) kits according to the manufacturers’ protocols (8-OHdG Check ELISA Kit, Cosmo Bio Ltd., Tokyo, Japan; 8-Isoprostane ELISA Kit, Cayman Chemical, Ann Arbor, MI, USA) [29]. According to the datasheets, the analytical detection range was 0.5–200 ng/mL for 8-OHdG and 0.8–500 pg/mL for 8-isoprostane, with a sensitivity of approximately 3 pg/mL for the latter. All reagents were stored at 2–8 °C, and analyses were carried out at 450 nm following incubation at 37 °C and color development in the dark. Creatinine concentration (mg/dL) was determined colorimetrically (Creatinine Assay Kit, Cayman Chemical, Ann Arbor, MI, USA), and biomarker values were normalized for urinary dilution and expressed as ng of biomarker per mg of creatinine, as previously described [29]. All assays were performed blinded to case–control status. Intra- and inter-assay variability were within the ranges reported by the manufacturers for research-use kits. Data integrity and distributional assumptions were verified prior to analysis. The National Institute of Cancer Research laboratory served as the single reference laboratory for all analyses to ensure methodological consistency and minimize inter-laboratory variability.

To obtain both total and differential WBC counts, the blood samples were immediately processed by hematology analyzer ADVIA 2120 (Siemens, Nürnberg, Germany).

### Food frequency questionnaire

The participants completed the EPIC questionnaire [26]. The daily intake of individual foods (g/day), energy intake (EI) (kcal/day), macro and micronutrients were estimated using the Nutritional Analysis of Food Frequency Questionnaires, Milan, Italy) software [30].

The ratio of EI to the basal metabolic rate (BMR) was calculated for all the subjects; BMR was estimated using age and sex-specific equations [31].

We excluded subjects: (1) who did not fill in more than 20% of the questions of the FFQ.; (2) with values exceeding the cut points at the top and bottom 0.5% of the distribution of EI: BMR; (3) with extreme low (< 600 Kcal for women and < 800 kcal for men) or high (> 6000 Kcal for women and > 8000 kcal for men) levels of EI.

### Definition of cases and controls

**Fig. 1:**
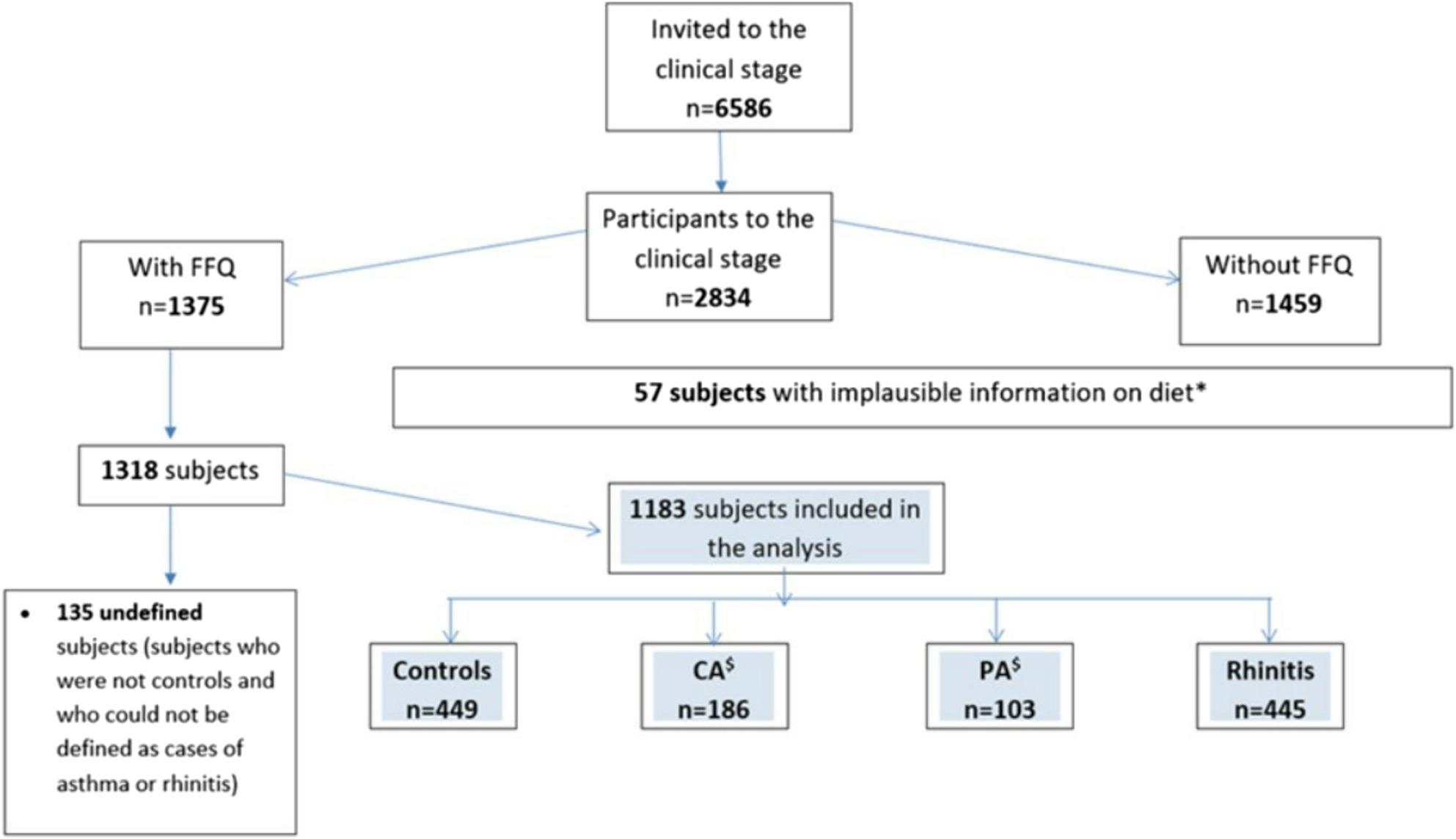
Flowchart of subjects’ selection (Ancona, Pavia, Sassari, Turin, Verona). Number of the subjects who were invited to the clinical stage, who took part in the GEIRD study clinical stage and who were considered in this work. *Exclusion criteria: •Incomplete FFQ (more than 20% of the EPIC questionnaire missing). •Subjects excluded according to the cut points based on the top and bottom 0.5% of the distribution of EI: BMR. •Subjects with extremely low levels (<600 Kcal for women and <800 kcal for men) or high levels (>6000 Kcal for women and >8000 Kcal for men) of EI. ^$^CA: current asthma, PA: past asthma

The number of subjects invited to clinics was 6586, of whom 2834 (43.03%) attended the clinical stage and 1375 (48.52%) filled in the FFQ. Fifty-seven subjects with implausible information on diet were excluded and the remaining 1318 subjects were classified according to the status of control/case. 135 subjects could not be classified due to missing information on clinical questionnaires/tests (Fig. 1).

The hierarchical classification included 1183 subjects (Fig. 1), classified as follows:

- 186 cases of current asthma (CA), described by one of the following definitions:
  - self-reported asthma and asthma-like symptoms in the last 12 months/current use of asthma medications.
  - self-reported asthma or asthma-like symptoms in the last 12 months/current use of asthma medications plus one among one of the following conditions: (a) a positive methacholine challenge test (PD20 ≤1 mg); (b) pre-bronchodilator FEV1/FVC < 70 % or <LLN with a positive reversibility test (FEV1> 12 % and >200 ml after 400 μg salbutamol by inhalation);(c) pre-bronchodilator FEV1/FVC < 70 % or <LLN with a post-bronchodilator FEV1/FVC > LLN and >70 % and a post-bronchodilator FEV1> 80 % predicted;
- 103 cases of past asthma (PA), self-reported history of asthma without the criteria for CA;
- 445 cases of rhinitis (RN) in absence of asthma defined as: (a) lifetime nasal allergies; (b) lifetime problem with sneezing, or a runny or blocked nose (without cold/flu); (c) recurrent nasal/eye symptoms in the presence of dust, pollens, or animals;
- 449 controls, subjects who were not cases, who did not report other respiratory symptoms or diseases, and with FEV1/FVC >=70% and >=LLN and FEV1 >80% predicted.

### Mediterranean diet scores and Mediterranean diet adherence

The MD adherence was assessed by the Italian Mediterranean Diet Index (IMI) [19, 20] and the Modified Mediterranean Diet Score (MDS) [21, 22] (Table 1s). Three categorical variables, each with three categories (low, medium, high) were created to describe MD adherence. For IMI, the adherence definition is found in Agnoli [19], but by combining the two categories at the bottom (0–1 and 2–3 joined in 0–3: low adherence; 4–5: medium adherence; 6–11: high adherence). For MDS, we applied the original categorization of Trichopoulou [32] (0–3: low adherence; 4–5: medium adherence; 6–9: high adherence).

### Statistical analysis

Subjects**’**characteristics were reported as percentages, median and interquartile range (IQR); nutrients and food intake were summarized as median and IQR. Differences among groups were tested by using the χ^2^ test and Kruskall-Wallis test, as appropriate.

To investigate the association between the MD adherence and case-control status, we fitted one multinomial regression model for each categorical variable describing adherence according to the scores, to the data. These models have four levels (control, RN, CA, and PA) for the dependent variable that defines the case-control status. The association of MD adherence with the case-control status was expressed through Relative Risk Ratios (RRR) (controls = reference category) and relative 95% CI. Alternative models replaced the categorical variable describing adherence with the corresponding MD score.

The potential confounders included center; gender; age; BMI, education, either continuous (age at which full-time studies were completed) or dichotomous (low : full-time studies completed at 16 years or below; high: full-time studies completed after the age of 16); smoking habits (never smoker; past smoker; current smoker); total energy intake (kcal/day); comorbidities (presenting at least one comorbidity among diabetes, cardiovascular diseases, cancer) and physical activity (heavy: almost 4–7 h of daily activity every day or almost 4–6 days of activity in a week; moderate: almost 1–3 h of daily activity every day or 1–3 days in a week; light: almost ½ hour of daily activity every day or one day in a month; no activity: none). In addition, we tested the two-way interaction terms of smoking habits and MD adherence and BMI and MD adherence one at a time.

To test for the linear trend across MD adherence levels, we treated the categorical variable defining adherence as a continuous one.

8-OHdG, 8-isoprostane and WBCs were considered as outcomes in multivariable quantile regressions. The adjusted regression b-coefficients, 95%CI and *p-values* for the association between the analyzed biomarkers and the MD adherence were estimated considering the controls, subjects with CA, PA and RN, in separate models. We reported differences in medians between level 2 (intermediate) and the reference level 1 (low) and between level 3 (high) and the reference level 1 (low) of adherence according to each MD score. The analyses were conducted using STATA version 17.

## Results

The main characteristics of the sample are presented in Table 1. Age was significantly different across groups (*p* < 0.001) with asthmatic subjects being younger than subjects with rhinitis and controls. FEV1% predicted and FEV1/FVC were different among groups (*p* < 0.001), with the lowest values observed in CA and the highest in controls.

**Table 1:** Main demographic, lifestyle, and clinical characteristics of the population under study

	**Controls** **(** ***n*** **= 449)**	**Current Asthma (** ***n*** ** = 186)**	**Past Asthma (** ***n*** ** = 103)**	**Rhinitis only (** ***n*** ** = 445)**	***p*** value*
Age (years)^a^	51.7 (44.2;60.5)	48.1 (42.0;57.8)	45.2 (37.8;50.8)	50.0 (42.4;59.1)	**< 0.001**
Sex (%)					0.956
Females	51.9	52.2	52.4	53.7	
BMI^a^ (kg/m2)	25.0 (22.4;27.5)	25.0 (22.0;27.8)	23.6 (21.5;26.5)	24.5 (22.2;27.3)	0.158
Smoking habits (%)					0.227
Non-smokers	54.2	50.5	56.9	52.4	
Ex smokers	33.1	29.6	27.4	29.4	
Current smokers	12.7	19.9	15.7	18.2	
Caloric intake^a^ (kcal/day)	1894.4 (1483.5;2423.6)	1846.7 (1530.6;2327.2)	2199.8 (1652.1;2598.5)	1910.7 (1537.1;2421.8)	0.082
Physical activity (%)					0.351
Heavy	5.57	4.30	5.83	8.09	
Moderate	36.30	33.87	33.01	36.85	
Light	7.13	12.37	10.68	8.76	
No activity	51.00	49.46	50.49	46.29	
Educational level^a^ (age, years)	19 (17;23)	19 (16;23)	19 (18;24)	19 (19;23)	0.102
Comorbidities (%)					0.190
Yes	18.4	22.4	20.4	24.3	
Adherence based on IMI (%)					0.022
Low	29.2	39.2	28.2	33.3	
Medium	38.5	38.2	32.0	39.5	
High	32.3	22.6	39.8	27.2	
Adherence based on MDS (%)					0.339
Low	37.2	39.3	34.0	36.6	
Medium	45.9	50.5	54.4	48.8	
High	16.9	10.2	11.6	14.6	
	**Controls** **(** ***n*** **= 331)**	**Current Asthma (** ***n*** ** = 150)**	**Past Asthma (** ***n*** ** = 75)**	**Rhinitis only (** ***n*** ** = 338)**	
8-OHdG^b,^^a^ng/mg	3.6 (1.8;7.0)	3.8 (1.9;6.5)	4.3 (2.0;8.0)	4.0 (2.0;7.2)	0.529
	**Controls** **(** ***n*** **= 288)**	**Current Asthma (** ***n*** ** = 111)**	**Past Asthma (** ***n*** ** = 44)**	**Rhinitis only (** ***n*** ** = 263)**	
8-isoprostane ^a^ng/mg	0.85 (0.4;1.8)	0.95 (0.5;1.8)	0.92 (0.4;2.5)	0.82 (0.4;1.6)	0.680
	**Controls** **(** ***n*** **= 449)**	**Current Asthma (** ***n*** ** = 181)**	**Past Asthma (** ***n*** ** = 95)**	**Rhinitis only (** ***n*** ** = 425)**	
FEV1% predicted^c^	102.7 (94.6;111.4)	92.2 (83.0;103.4)	98.6 (93.5;107.0)	101.6 (93.8;110.9)	**< 0.001**
FVC % predicted^c^	97.6 (84.5;118.2)	95.0 (82.6;115.1)	94.6 (84.1;114.6)	98.9 (85.1;115.6)	0.486
FEV1/FVC^c^	81.7 (77.6;85.4)	75.9 (69.5;80.2)	80.4 (77.3;83.7)	81.0 (77.8;84.8)	**< 0.001**
	**Controls** **(** ***n*** **= 426)**	**Current Asthma (** ***n*** ** = 177)**	**Past Asthma (** ***n*** ** = 101)**	**Rhinitis only (** ***n*** ** = 422)**	
Atopy (%)					**< 0.001**
Yes	23.5	76.3	71.3	56.9	

^a^median (p25-p75)

^b﻿^8-hydroxy-2'-deoxyguanosine

^c^﻿Lung function values were expressed as a percentage of predicted value

^*^p values were calculated using the Kruskal-Wallis test for continuous variables and χ2 test for categorical ones

**Fig. 2:**
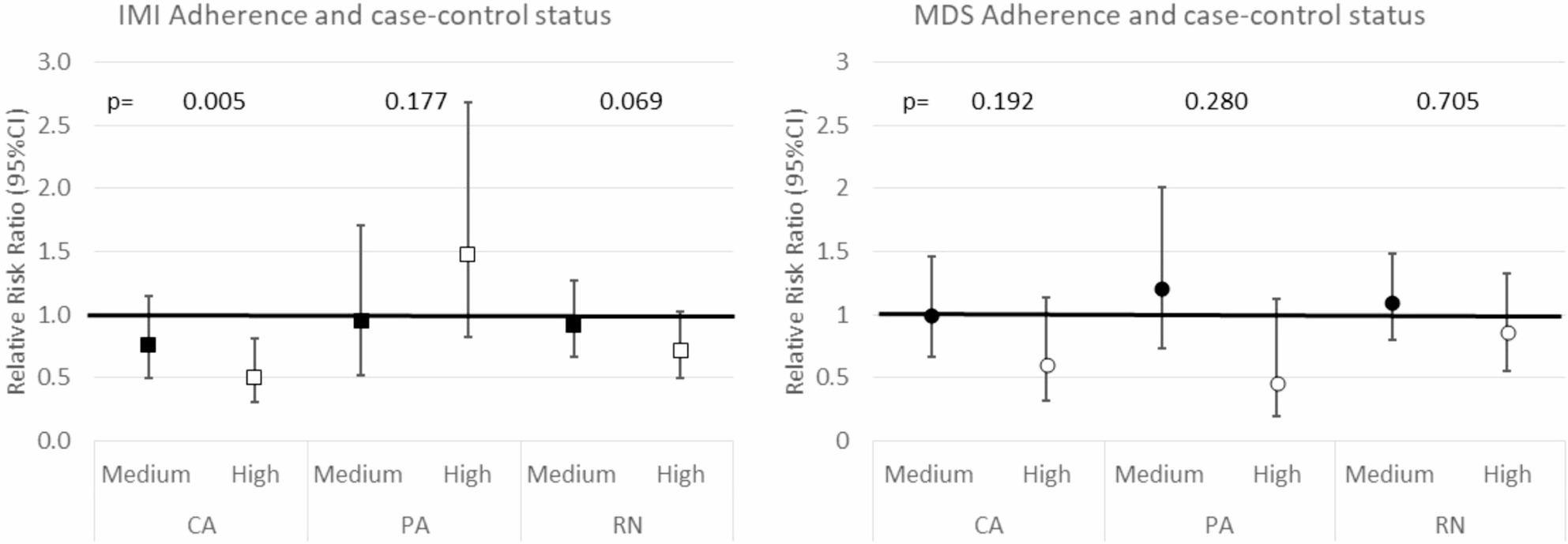
Adjusted RRR (95%CI) to be a case according to the adherence based on IMI and MDS and *p*-values for trend. The reference category is level 1 of the adherence variable-i.e. low adherence-for IMI and MDS in the GEIRD study. The estimates were adjusted for age, gender, BMI, smoking habits, educational level, total energy intake study, physical activity, comorbidities and center

In the multivariable analysis, the subjects with the highest MD adherence, as measured by IMI, had a lower RRR of being CA compared to controls (Fig. 2). Specifically, the risk of being a CA decreases by 50% in subjects at the highest compared to lowest MD adherence (*p* = 0.005). This association was consistent with the results obtained using the IMI-MD score as a quantitative covariate (RRR = 0.86; 95%CI:0.78;0.95: for every one-point increase in the IMI score the risk of being a subject with CA rather than a control decreased by 0.86 times) and with the *p-value* for trend of MD adherence by the IMI (*p* = 0.005). In contrast, MD adherence according to MDS was not associated with the case-control status, although a protective, but not statistically significant effect of high MD adherence on CA was observed (RRR = 0.60, 95%CI: 0.32;1.13, *p* = 0.112) (Fig. 2). When considering the MDS-MD score as a quantitative covariate, the association was still not significant (RRR = 0.88; 95%CI:0.76;1.02; *p* = 0.088).

**Fig. 3:**
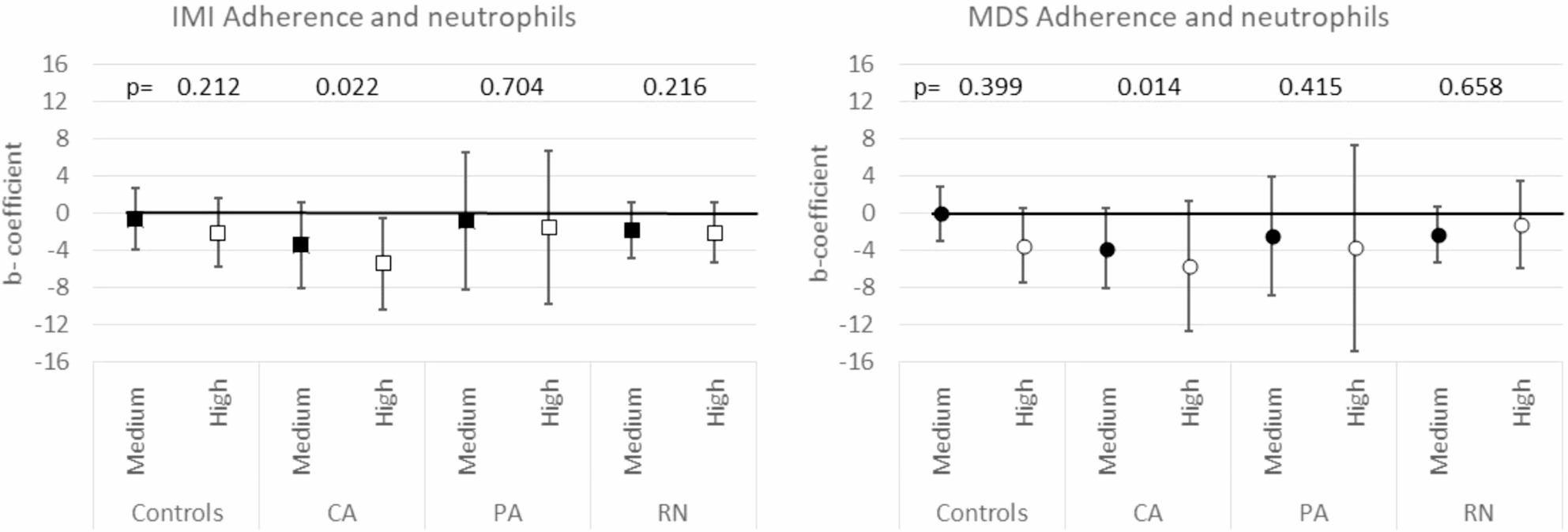
Adjusted multivariable quantile regression b-coefficients (95%CI) and p-values for trend for neutrophils. For each set of cases and controls, the b-coefficient estimates were derived separately. For each score they represent the neutrophils difference in medians with respect to the reference level (% of neutrophils out of total leucocytes), between level 2 (medium) and the reference level 1 (low) and between level 3 (high) and the reference level 1 (low) adherence. The estimates were adjusted for age, gender, BMI, smoking habits, educational level, physical activity, total energy intake, comorbidities and center

Multivariable quantile regression revealed no associations between 8-OHdG, 8-isoprostane and the MD adherence, based either on MDS or IMI, in most of the groups of cases and controls (Tables 2 and 3). However, in subjects with PA, those with a high adherence level according to MDS showed a median level of 8-isoprostane 5.30 ng/mg_creat_ lower than those in the low level of adherence (*p* = 0.026) and in subjects with CA, those with a high MD adherence level according to MDS showed a median level of 8-OHdG 2.70 ng/mg_creat_ higher than those in the low level of adherence (*p* = 0.040). Moreover, in CA subjects, a higher level of IMI-MD adherence was associated with a lower percentage of neutrophils out of total leucocytes: CA with high adherence had a 5.44 lower percentage of neutrophils than those with a low IMI-MD adherence level (*p* = 0.031) (Fig. 3). A significant trend from low to high IMI-MD adherence was also observed (*p* = 0.022), with a lower neutrophil percentage in the high adherence group. In the same group, a statistically significant descending trend between MDS–MD adherence and neutrophil percentage was found (*p* = 0.014). (Fig. 3). In controls, the subjects with a high level of IMI-MD adherence had a 0.48 lower eosinophil percentage than those with a low level of IMI-MD adherence, even if the difference was not statistically significant (*p* = 0.063) (Table 3s). In the analyses of total leucocytes and lymphocytes, we did not find any relevant association (Tables 2s-4s).

**Table 2:** Adjusted multivariable quantile regression b-coefficients for the outcome 8-OHdG

		IMI				MDS			
	**Adherence levels**	Coefficient^a^	**95% CI**	***P value***	***P trend***	Coefficient^a^	**95% CI**	***P value***	***P trend***
Controls	Medium	0.07	-1.26;1.40	0.917	0.575	-0.72	-1.93;0.49	0.240	0.467
	High	-0.38	-1.81;1.04	0.596		-0.40	-2.12;1.32	0.646	
Current Asthma	Medium	1.00	-0.66;2.66	0.237	0.782	0.50	-0.94;1.95	0.490	0.073
	High	0.16	-1.75;2.08	0.866		2.70	0.13;5.28	0.040	
Past Asthma	Medium	1.13	-3.13;5.39	0.598	0.376	-0.02	-3.46;3.41	0.990	0.925
	High	-0.40	-5.00;4.21	0.863		-1.01	-8.21;6.19	0.780	
Rhinitis only	Medium	0.85	-0.46;2.16	0.201	0.229	0.87	-0.44;2.18	0.191	0.324
	High	0.94	-0.56;2.44	0.218		0.95	-0.98;2.87	0.333	

For each set of cases and controls, the estimates were derived separately. For each score, the regression coefficients represent the 8-hydroxy-2'-deoxyguanosine (8-OHdG) difference in medians with respect to the reference level (ng/mg_creat_), between level 2 (medium) and the reference level 1 (low) and between level 3 (high) and the reference level 1 (low) of adherence. The estimates were adjusted for age, gender, BMI, smoking habits, educational level, total energy intake, physical activity, comorbidities and center

^a^Difference in medians with respect to the reference level (ng/mg_creat_)

**Table 3:** Adjusted multivariable quantile regression b-coefficients for the outcome 8-isoprostane

		IMI				MDS			
	**Adherence levels**	Coefficient^a^	**95% CI**	***P value***	***P trend***	Coefficient^a^	**95% CI**	***P value***	***P trend***
Controls	Medium	0.01	-0.30;0.32	0.951	0.989	-0.02	-0.31;0.27	0.887	0.646
	High	-0.01	-0.34;0.32	0.954		-0.08	-0.49;0.33	0.698	
Current Asthma	Medium	-0.15	-0.68;0.39	0.589	0.099	0.13	-0.40;0.67	0.623	0.838
	High	-0.56	-1.19;0.08	0.085		-0.39	-1.37;0.59	0.435	
Past Asthma	Medium	0.02	-2.63;2.67	0.990	0.994	-0.56	-1.96;0.83	0.414	0.030
	High	-0.14	-2.63;2.36	0.912		-5.30	-9.91;-0.70	0.026	
Rhinitis only	Medium	0.20	-0.14;0.55	0.243	0.665	-0.09	-0.43;0.26	0.625	0.590
	High	0.10	-0.29;0.49	0.619		-0.05	-0.54;0.45	0.845	

For each set of cases and controls, the estimates were derived separately. For each score, they represent the 8-isoprostane difference in medians with respect to the reference level (ng/mg_creat_), between level 2 (medium) and the reference level 1 (low) and between level 3 (high) and the reference level 1 (low) of adherence. The estimates were adjusted for age, gender, BMI, smoking habits, educational level, total energy intake, physical activity, comorbidities and center

^a^Difference in medians with respect to the reference level (ng/mg_creat_)

## Discussion

In this study on a sample of adults from the general population, a higher adherence to a healthy MD was associated with a lower CA, but not with PA or RN prevalence. The adherence to MD was negatively related to the number of circulating neutrophils, the first WBCs involved in the inflammatory response, only in CA. Urinary 8-isoprostane was not associated with the adherence to MD in any group, whereas a positive association was found between MD and 8-OHdG urinary concentrations, only in CA. The association between MD adherence and a reduced prevalence of CA, but not of PA and RN, suggests that MD may reduce respiratory symptoms, which are one of the key elements of the definition of CA in the present study, without influencing the risk of having had asthma in the past. This finding aligns with previous studies, reporting that higher MD adherence is associated with a lower asthma symptom score in adults [33] and with the likelihood of asthma being under control in a small adult outpatients’ asthmatic cohort [11]. Several scores (other than those related to the MD), such as the Alternate Healthy Eating Index-2010, have been associated with better asthma outcomes in middle-aged adults [33, 34] and elderly women [35]. While our study suggests a beneficial association between MD and CA, it is unknown whether an intervention to promote the MD could reduce the prevalence of asthma or symptoms, since there are no RCTs testing this hypothesis in adults. Our data gives no proof of whether MD could be a protective factor for asthma everywhere or just in some type of environments such the Mediterranean basin [15] or whether the association between MD and CA decreased prevalence might be linked to the co-occurrence of socioeconomic, behavioral, and lifestyle characteristics which promotes healthier habits, such as physical exercise or BMI control [32]. Of note, all the aforementioned factors were considered in the multivariate analysis, supporting the hypothesis that diet is independently associated to CA. The lack of a link between the MD and rhinitis indirectly supports previous findings, reporting that high MD compliance was not protective against allergic rhinitis in children [36].

Greater MD adherence is consistently associated with reduced chronic diseases, cancer risk [37, 38] and overall mortality [39]. The leading hypothesis on the mechanism of this association is the high MD antioxidant capacity [40]. However, both observational and intervention studies exploring the associations between MD and oxidative stress biomarkers yielded contradictory results [41]. We found no relevant relationship between MD adherence and urinary 8-isoprostane, suggesting no crucial modulation of the lipid peroxidation of arachidonic acid, of which 8-isoprostane is a product [42], and that 8-isoprostane is not involved in the possible protective mechanisms of MD against CA. Previous reports also provided inconclusive results, some of them suggesting a negative association between MD and F2-isoprostane [43, 44], others no relationship [45, 46]. To the best of our knowledge, this is the first study investigating the association between MD and markers of oxidative DNA damage in a general population sample. Intriguingly, we found a positive association between MD adherence and urinary 8-OHdG in CA subjects. This is in contrast with previous intervention studies, suggesting a role of diet in decreasing 8-OHdG in DNA extracted from peripheral leucocytes [47] and in reducing its urinary/plasma concentrations [47, 48]. However, most of the above-mentioned studies did not focus on the MD role as such, but in combination with bioactive substances [47–49], making a specific MD contribution difficult to determine; furthermore, they were carried out on selected groups of patients [48–50] or elderly people [48] dissimilar to our general population sample. The underlying mechanisms of this observed positive relationship remains unclear, as certain MD components such as tomatoes or shellfish could contribute to DNA oxidative damage [46, 51].

The association between MD and the urinary 8-OHdG was only significant in CA and when MD adherence was measured by MDS, confirming that a residual confounding due to the characteristics of the population studied, the biomarkers measured and the method used to assess adherence or chance may play a role in defining the association between the quality of diet and oxidative stress.

As previously mentioned, diet is a composite exposure variable, meaning that the use of dietary patterns, rather than single nutrients, is a more suitable method of investigation [4]. For example, systematic reviews highlight the strong inverse associations between high adherence to the Mediterranean diet and a wide range of chronic outcomes [52]. Moreover, it has been shown that Mediterranean-diet indices reveal more consistent associations for pattern scores than for single nutrients or isolated foods [53]. In this regard, various MD scores have been formulated and used in research, including the MDS and the IMI. Our analysis shows that the use of these two scores does not always provide overlapping results, probably because they refer to different settings and therefore to some different food components (Table 1S): the IMI is specific for the Italian diet, while the MDS is the original method for scoring MD adherence in the Hellenic area.

Dietary patterns rich in fruits and vegetables, whole grains, and fish, such as the MD, are often linked to lower systemic inflammation [16]. An increased WBC count is a broadly used marker of systemic inflammation [54], and prior research tried to link it to dietary patterns [55–57]. Previous reports have shown that higher MD compliance assessed by MDS and IMI is inversely associated with total WBC [57]. In partial contrast to this, our findings show that a greater MD adherence is not significantly associated with a reduction in the total WBC count in any group. However, WBC subgroup analyses revealed an inverse relationship between the higher MD adherence and neutrophil count (the first WBCs involved in the inflammatory response [58]), in CA but not in PA, RN or in controls. Of note, eosinophils, considered a key factor in asthma pathophysiology [59], were not associated with the investigated scores. These findings suggest that MD adherence is not associated with a reduction in inflammation in all subjects and illnesses, but only in specific diseases and that only neutrophils seem to be involved in this mechanism. The role of neutrophils in asthma is not fully understood [60], even though there are data supporting that airway neutrophilia is associated with worse outcomes, such as lung function impairment and increased hospitalizations [61]. It is worth noting that neutrophilic asthma has a low prevalence in Greece, where the MD adherence is presumably high [60]. Also of interest are the findings of Douros et al. [62], who found an inverse association in asthmatic children between MD adherence and IL-17, a cytokine implicated in neutrophilic asthma pathogenesis [63]. These observations could suggest a positive effect of MD adherence in reducing circulating neutrophils and possibly the extent of chronic low-grade inflammation.

Previous reports hypothesized that gut microbiota might play a role in the diet-asthma relationship, probably through the production of short-chain fatty acids (SCFAs) [64], the end products of fermentation of dietary fibers in the gut known to be associated with a diet characterized by a high content of fruit, vegetables and legumes [65]. In animal models, both dietary fiber and SCFAs have demonstrated anti-inflammatory effects via the activation of free fatty acid receptors [66]. Furthermore, it has recently been reported that a soluble fiber meal administered to adults with stable asthma can reduce airway inflammation biomarkers in sputum, including neutrophils [67]. Based on these premises, we speculate that SCFAs as components of MD may reduce inflammation due to circulating neutrophils, although further studies are needed to support this hypothesis.

The main strengths of this study include its general population sample and the use of standardized protocols both for dietary assessment [26] and for the accurate identification of cases of respiratory diseases. Moreover, we investigated the association between MD adherence and respiratory diseases and between MD adherence and oxidative stress biomarkers in the same population, simultaneously assessing the potential clinical effect and the underlying mechanism. The potential limitation could be the cross-sectional nature of our case-control study that does not allow us to make inferences on the causal effects. Therefore, we cannot exclude that our results may be due to reverse causation due to the change of diet after diagnosis. However, this is unlikely, as there were no explicit indications on Mediterranean diet in guidelines for subjects with asthma at the time of the study. In addition, we explored several hypotheses on the association between diet and oxidative stress or inflammation biomarkers in different group of cases and controls: we cannot exclude that this could have produced some significant association only by chance. Another limitation is that the recall bias may have affected the FFQ fulfilment. However, as a mitigation measure, subjects with a poor-quality dietary data were excluded and there is evidence that adults maintain relatively stable long-term dietary habits [68, 69].

In conclusion, our findings indicate that MD is associated with lower odds of CA, although more investigations are required to establish whether this association is causative. In contrast, the MD compliance does not seem to influence RN or PA prevalence. It would be possible that MD reduces inflammation in CA by decreasing the number of circulating neutrophils, whereas oxidative stress, measured by 8-OHdG and 8-isoprostane, does not appear to play a significant role. Given the healthcare burden resulting from asthma management, clinicians may consider incorporating dietary history and possible dietary changes to promote comprehensive respiratory care and generate a complementary benefit in general health.

## Supplementary Information

Supplementary Material 1.

## Data Availability

The datasets analysed during the current study are not publicly available as they belong to the Steering Committee of the GEIRD study, but are available from the corresponding author on reasonable request.

## Abbreviations

8-isoprostane: 8-iso-prostaglandin F_2α_
8-OHdG: 8-hydroxy-2'-deoxyguanosine
ATS: American Thoracic Society
BMI: Body mass index
BMR: Basal metabolic rate
CA: Current asthma
CI: Confidence interval
EI: Energy intake
EPIC: European Investigation Into Cancer and Nutrition
FEV1: Forced expiratory volume in 1 s
FFQ: Food frequency questionnaire
FVC: Forced vital capacity
GEIRD: Genetic and Environmental interaction in Respiratory Diseases
IMI: Italian Mediterranean Index
IQR: Interquartile range
MD: Mediterranean diet
MDS: Modified Mediterranean Score
PA: Past asthma
PD20: Provocative dose causing a 20% decrease in FEV1
RCT: Randomized clinical trial
RN: Rhinitis
RRR: Relative risk ratios
SCFAs: Short-chain fatty acids
LLN: Lower limit of normal
WBC: White blood cells
