# Supplementary material for: Mediterranean diet and asthma in adults: a multicentre case-control study in a general population sample
Source: Respir Res. 2026 Jan 13;27:49. doi: 10.1186/s12931-025-03484-3 (PMC12888413; doi:10.1186/s12931-025-03484-3)
Supplement: Supplementary file 1 — Supplementary Material 1. [file 12931_2025_3484_MOESM1_ESM.docx]

Supplement materials

Table 1S. Mediterranean diet scoring system food components.

|  | **IMI**^a^  (Italian Mediterranean score) | **MDS**^b^  (Modified Mediterranean Diet Score) |
| --- | --- | --- |
| **Components** |  |  |
| **Mediterranean foods** | | |
| Cereals ^c^ | 1 | 1 |
| Total vegetables^d^ | - | 1 |
| Mediterranean vegetables^e^ | 1 | - |
| Olive oil | 1 | - |
| MUFAs/SFA^f^ | - | 1 |
| Fresh fruit | 1 | - |
| All fruit and juices | - | - |
| Fruits and nuts | - | 1 |
| Nuts | - | - |
| Legumes | 1 | 1 |
| Fish | 1 | 1 |
| Alcohol | 1^g^ | - |
| **Not-typically-Mediterranean foods** | | |
| Potatoes | 1 | 1 |
| Dairy products | - | 1 |
| Meat and meat products | 1 | 1 |
| Butter | 1 | - |
| Soft drink | 1 | - |
|  |  |  |
| **Score range** | 0-11 | 0-9 |

^a^ 1 point for partecipants in the third tertile of consumption of Mediterranean foods and 1 point for subjects in the first consumption tertile of other not-typically-Mediterranean foods.

^b^ one point is assigned to partecipants whose consumption of Mediterranean foods exceeds the sex-specific median, while an additional point is given to sbjects whose consumption of other non-typically-Mediterranean foods is below the sex-specific median.

^c^ Pasta for IMI; Cereals for MDS.

^d^ Excluding potatoes.

^e^ Raw tomatoes, non-brassica leafy vegetables, courgettes, aubergines, peppers, onion, garlic, salad, fruiting vegetables.

^f^ mono-unsaturated fatty acids to saturated fatty acids (MUFAs-to-SFAs) ratio.

^g^ 1 point for intake up to 12g/day, abstainers and intake>12g/day 0 points.

Table 2s. Adjusted multivariable quantile regression b-coefficients for the outcome **lymphocytes**.

|  |  | **IMI** |  |  |  | ***MDS*** |  |  |  |
| --- | --- | --- | --- | --- | --- | --- | --- | --- | --- |
|  | **Adherence levels** | **Coefficient**† | **95% CI** | ***P value*** | ***P trend*** | **Coefficient**† | **95% CI** | ***P value*** | ***P trend*** |
| **Controls** | **Medium** | 0.33 | -2.67;3.32 | 0.830 | 0.293 | 0.25 | -2.46;2.96 | 0.854 | 0.408 |
|  | **High** | 1.17 | -1.63;5.05 | 0.313 |  | 1.99 | -1.65;5.63 | 0.282 |  |
| **Current Asthma** | **Medium** | 2.73 | -0.83;6.29 | 0.130 | 0.024 | 3.10 | -0.13;6.32 | 0.059 | 0.477 |
|  | **High** | 3.69 | -0.16;7.54 | 0.060 |  | 1.25 | -3.94;6.44 | 0.633 |  |
| **Past Asthma** | **Medium** | 2.13 | -3.32;7.58 | 0.434 | 0.220 | 3.68 | -2.04;9.39 | 0.201 | 0.176 |
|  | **High** | 4.93 | -1.16;11.01 | 0.110 |  | 5.20 | -4.68;15.07 | 0.294 |  |
| **Rhinitis only** | **Medium** | 0.28 | -2.40;2.96 | 0.838 | 0.597 | 2.19 | -0.27;4.65 | 0.081 | 0.194 |
|  | **High** | 1.20 | -1.77;4.17 | 0.426 |  | 1.80 | -2.03;5.62 | 0.355 |  |

For each set of cases and controls, the estimates were derived separately. For each score, they represent the **Lymphocytes** difference in medians with respect to the reference level (% of lymphocytes out of total leucocytes), between level 2 (medium) and the reference level 1 (low) and between level 3 (high) and the reference level 1 (low) adherence. The estimates were adjusted for age, gender, BMI, smoking habits, educational level, total energy intake, physical activity, comorbidities and center.

† Difference in medians with respect to the reference level (% of lymphocytes out of total leucocytes)

Table 3s. Adjusted multivariable quantile regression b-coefficients for the outcome **eosinophils**.

|  |  | **IMI** |  |  |  | ***MDS*** |  |  |  |
| --- | --- | --- | --- | --- | --- | --- | --- | --- | --- |
|  | **Adherence levels** | **Coefficient**† | **95% CI** | ***P value*** | ***P trend*** | **Coefficient**† | **95% CI** | ***P value*** | ***P trend*** |
| **Controls** | **Medium** | -0.24 | -0.69;0.22 | 0.309 | 0.063 | 0.35 | -0.09;0.79 | 0.118 | 0.812 |
|  | **High** | -0.48 | -0.99;0.03 | 0.063 |  | -0.13 | -0.72;0.46 | 0.671 |  |
| **Current Asthma** | **Medium** | 0.49 | -0.74;1.72 | 0.432 | 0.306 | 0.48 | -0.67;1.63 | 0.409 | 0.639 |
|  | **High** | 0.66 | -0.68;1.99 | 0.328 |  | 0.05 | -1.80;1.91 | 0.956 |  |
| **Past Asthma** | **Medium** | 0.56 | -0.43;1.55 | 0.256 | 0.342 | 0.10 | -0.78;0.97 | 0.826 | 0.761 |
|  | **High** | 0.82 | -0.29;1.92 | 0.142 |  | 0.06 | -1.45;1.56 | 0.941 |  |
| **Rhinitis only** | **Medium** | 0.01 | -0.48;0.51 | 0.958 | 0.211 | -0.04 | -0.53;0.45 | 0.868 | 0.779 |
|  | **High** | -0.33 | -0.88;0.21 | 0.232 |  | -0.15 | -0.91;0.62 | 0.705 |  |

For each set of cases and controls, the estimates were derived separately. For each score, they represent the **Eosinophils** difference in medians with respect to the reference level (% of eosinophils out of total leucocytes) between level 2 (medium) and the reference level 1 (low) and between level 3 (high) and the reference level 1 (low) adherence. The estimates were adjusted for age, gender, BMI, smoking habits, educational level, physical activity, total energy intake, comorbidities and center.

† Difference in medians with respect to the reference level (% of eosinophils out of total leucocytes).

Table 4s. Adjusted multivariable quantile regression b-coefficients for the outcome **leucocytes**.

|  |  | **IMI** |  |  |  | **MDS** |  |  |  |
| --- | --- | --- | --- | --- | --- | --- | --- | --- | --- |
|  | **Adherence levels** | **Coefficient**† | **95% CI** | ***P value*** | ***P trend*** | **Coefficient**† | **95% CI** | ***P value*** | ***P trend*** |
| **Controls** | **Medium** | -0.19 | -0.76;0.38 | 0.509 | 0.301 | -0.23 | -0.71;0.26 | 0.358 | 0.100 |
|  | **High** | -0.33 | -0.97;0.30 | 0.305 |  | -0.59 | -1.24;0.06 | 0.077 |  |
| **Current Asthma** | **Medium** | 0.70 | -0.47;1.87 | 0.237 | 0.918 | -0.70 | -1.85;0.45 | 0.230 | 0.290 |
|  | **High** | -0.42 | -1.62;0.78 | 0.487 |  | -1.13 | -2.98;0.72 | 0.199 |  |
| **Past Asthma** | **Medium** | -0.83 | -2.39;0.74 | 0.293 | 0.879 | 0.69 | -0.77;2.16 | 0.344 | 0.144 |
|  | **High** | -0.33 | -2.08;1.42 | 0.707 |  | 1.78 | -0.75;4.31 | 0.163 |  |
| **Rhinitis only** | **Medium** | -0.29 | -0.92;0.35 | 0.377 | 0.764 | -0.06 | -0.68;0.56 | 0.853 | 0.922 |
|  | **High** | -0.15 | -0.85;0.56 | 0.683 |  | -0.02 | -0.98;0.94 | 0.973 |  |

For each set of cases and controls, the estimates were derived separately. For each score, they represent the **Leucocytes** difference in medians with respect to the reference level (n*10^3^/µl), between level 2 (medium) and the reference level 1 (low) and between level 3 (high) and the reference level 1 (low) adherence. The estimates were adjusted for age, gender, BMI, smoking habits, educational level, physical activity, total energy intake, comorbidities and center.

† Difference in medians with respect to the reference level (n*10^3^/µl).
